# Gambling Problems Are Associated with Alcohol Misuse and Insomnia: Results from a Representative National Telephone Survey

**DOI:** 10.3390/ijerph18136683

**Published:** 2021-06-22

**Authors:** Hannah Briony Thorne, Matthew Justus Rockloff, Sally Anne Ferguson, Grace Elizabeth Vincent, Matthew Browne

**Affiliations:** School of Health, Medical and Applied Sciences, Central Queensland University, Adelaide 5034, Australia; m.rockloff@cqu.edu.au (M.J.R.); sally.ferguson@cqu.edu.au (S.A.F.); g.vincent@cqu.edu.au (G.E.V.); m.browne@cqu.edu.au (M.B.)

**Keywords:** problem gambling, alcohol misuse, hazardous drinking, sleep problems, insomnia, sleep restriction

## Abstract

Gambling has significant costs to the community, with a health burden similar in scale to major depression. To reduce its impact, it is necessary to understand factors that may exacerbate harm from gambling. The gambling environment of late-night licensed venues and 24/7 online gambling has the potential to negatively impact sleep and increase alcohol consumption. This study explored gambling, alcohol, and sleep problems to understand whether there is a relationship between these three factors. Telephone interviews were conducted with a representative sample of Australian adults (n = 3760) combined across three waves of the National Social Survey. Participants completed screening measures for at-risk gambling, at-risk alcohol consumption, insomnia (2015 wave only), and sleep quality. There were small but significant positive correlations between problem gambling and alcohol misuse, problem gambling and insomnia, and problem gambling and poor sleep quality. A regression model showed that gambling problems and alcohol misuse were significant independent predictors of insomnia. A separate regression showed gambling problems (and not alcohol misuse) were a significant predictor of poor sleep quality, but only in one survey wave. Findings suggest that gambling, alcohol, and sleep problems are related within persons. Further research should examine the mechanisms through which this relationship exists.

## 1. Introduction

Gambling is a popular leisure activity that is easily accessed in many countries, particularly across the Western world [1,2,3,4]. However, despite its legality and its continuing proliferation, attendant gambling harms have been associated with a reduction in health-related quality of life [5]. Moreover, people with severe gambling problems have been shown to bear a health burden of similar scale to major depressive disorder [5]. Recent research has also highlighted the pervasiveness of harm from gambling across many aspects of people’s lives, including familial relationships, psychological well-being, and education [6,7]. To reduce the negative impact of gambling, it is necessary to understand the factors, or combinations of factors, that may exacerbate or contribute to the risk of experiencing gambling harm. One such combination of factors is alcohol consumption—specifically misuse—and sleep, in particular, inadequate sleep concomitant with gambling. Each of these factors may be precipitated by gambling options in late-night licenced venues and the availability of 24/7 mobile betting applications [8,9].

The association between alcohol consumption and gambling problems is well established. General population studies and studies of treatment-seeking gamblers have demonstrated high levels of comorbidity between problem gambling and alcohol misuse [4,10,11,12,13]. Comorbid problem gambling and alcohol misuse also have an impact on the amount of harm experienced [14]—for example, pathological gamblers that misuse alcohol are less likely to show improvements in problem gambling status over time when compared to those that do not misuse alcohol [15]. There is also a known relationship between sleep problems and alcohol misuse, with chronic misuse of alcohol negatively affecting sleep through changes in sleep structure [16]. Alcohol-use disorder is also a common comorbidity of insomnia, with this relationship exacerbating insomnia symptoms [16,17,18,19,20].

The link between gambling and sleep behaviours is less well documented. Loft and Loo [21] examined a small sample (n = 59) of treatment-seeking gamblers and found a significant positive correlation between the problem gambling severity index (PGSI, [22]) score and sleep difficulty, as well as between the PGSI score and negative sleep habits (e.g., unhealthy sleep practices). A large population study (n = 3435) based in the United States also reported that people experiencing problems with gambling were significantly more likely to report any, and all, of three insomnia criteria (difficulty initiating sleep, difficulty maintaining sleep, and early morning awakening) [23]. However, these studies have several limitations. Loft and Loo’s [21] sample was very homogeneous, being 95% male and almost entirely of Chinese ethnicity. This may have skewed their results as males often participate in different types of gambling than females [24,25] and are overly represented in problem gambling statistics [4,26]. Chinese samples show rates of problem gambling up to three times that of Western populations [27,28,29], demonstrating culture-specific gambling behaviours in Chinese people that may set them apart from other ethnic groups [30,31]. Parhami’s [23] population study also has an important limitation in that the data was collected between 2001 and 2003, prior to the influx of internet and mobile-based gambling. This expansion of online gambling has increased the accessibility of gambling during the late-night hours [32], potentially impacting sleep behaviours.

Few studies in the literature specifically examine the three factors of gambling, alcohol consumption, and sleep in the same population. Hamel et al. [33] explored the concept of “tilt”—a heightened emotional state resulting from in-game events, that can lead to poor decision making—and sleep deprivation in 23 (96% male) mainly Canadian (91%) poker players. Sleep deprivation (16 h or more had passed between awakening and the end of the gambling session) resulted in higher levels of tilt, and higher levels of tilt were also associated with increased alcohol consumption. Thorne et al. [34] examined the time course of gambling, alcohol consumption, and sleep over a six-day period using a United States–based online self-selected sample of 132 regular gamblers. They found a positive correlation over the six days between the amount of time spent gambling and the number of drinks consumed and a negative correlation between sleep duration and the number of drinks consumed, the amount of time spent gambling, and the duration of naps. However, they did not find any time-related day-by-day association between behaviours, only the co-occurrence of habits across persons. This may indicate that the time course of the study was too brief. Alternatively, there may be a third variable that mediates the relationship between sleep, gambling, and alcohol. It should be noted that while there are limitations related to online convenience samples [35], the aforementioned studies had rigorous checks in place to ensure data quality [33,34].

Australians lead the world in the amount of gambling losses sustained per capita, spending more than 60% more per person than Americans [36]. Australia arguably has one of the most liberal gambling markets in the world, especially regarding the proliferation of electronic gaming machines (EGMs) in many licensed pubs and clubs (Western Australia being an exception) [37]. It is also possible that the characteristics of samples used in past studies may mean results are not generalisable to the Australian population. For example, Hamel et al. [33] used a small group of online poker players, a group that has been shown to differ from those who gamble on more chance-based gambling modes [24]. This difference in jurisdictions and sample characteristics prompts the need to investigate whether the association between sleep and gambling is robust. The current study aimed to explore whether there are relationships between gambling, alcohol consumption, and sleep problems in an Australian adult population. A positive finding would justify future experimental or in situ studies that examine potential causal relationships between these variables.

## 2. Materials and Methods

### 2.1. Design

The 2015, 2016 (July–August), and 2016/2 (November) National Social Surveys were cross-sectional Computer Administered Telephone Interview (CATI) surveys conducted by the Population Research Laboratory (PRL) at Central Queensland University. The surveys focused on health behaviours, including smoking, drinking, exercise, gambling, and sleep. Ethical approval for each survey was received from the Human Research Ethics Committee at Central Queensland University (H14/09-203; H14/09-203; H14/09-203, in chronological order). All participants gave their verbal consent to participate prior to survey commencement.

### 2.2. Participants and Procedure

Telephone numbers were selected at random from national lists of both fixed and mobile numbers compiled by SampleWorx Pty Ltd. Sydney, Australia (SampleWorx), a reputable research sample provider. Known business numbers were excluded, and remaining numbers were silently pinged to ensure valid connections. Landline telephone numbers were chosen from the SampleWorx list at random but were stratified by postcodes to ensure roughly proportionate geographic representation relative to the population based on Australian 2011 census data. Mobile numbers were randomly generated prior to validation as valid. Mobile numbers are not assigned to customers by reliable location prefixes in Australia.

To sample mobile telephone numbers, if the person who answered the telephone call was aged over 18 and was currently residing in Australia, they were deemed eligible to participate in the survey. For landline telephone numbers, each household was designated as either a male or female household, based on random assignment to maintain gender balance. If the person who answered the telephone was not of the designated gender, the individual of the designated gender with the most recent birthday in the household was selected for the interview. If there was no one of the designated gender living in the household it was disqualified for use. To be eligible to participate, the household must have been the usual place of residence for the respondent, and they must have been over 18 years of age. Calls were made between 4:30 p.m. and 8:30 p.m. on weekdays; between 10:30 a.m. and 2:30 p.m. on Mondays, Wednesdays, and Fridays; and between 12:00 p.m. and 4:00 p.m. on weekends. Each telephone number received a minimum of five call-backs before they were designated as nonresponses. Table 1 shows the final sampling characteristics of the three datasets.

### 2.3. Measures

#### 2.3.1. Demographics

Standard demographic questions included in each wave of the National Social Survey include age, gender, state or territory of residence, country of birth, marital status, education level, and employment status. These measures are reported in Table 2.

#### 2.3.2. Gambling

The Lie/Bet [38] is a two-item screen for problem gambling. When tested against the DSM-IV criteria for pathological gambling, the Lie/Bet has been shown to have a positive predictive value of 0.92 and a negative predictive value of 0.99, being both sensitive and specific. The two items are (1) Have you ever had to lie to people important to you about how much you gamble? (2) Have you ever felt the need to bet more and more money? An affirmative answer to either question (or both) is scored as indicating problem gambling.

The Consumption Screen for Problem Gambling (CSPG, [39]) is a three-item gambling screen, based on time spent gambling. It has been shown to correctly identify severe cases of problem gambling indicated by the most commonly used longer form of problem gambling screening: the Problem Gambling Severity Index (PGSI, [22]). The CSPG consists of the following three questions: (1) How often did you gamble in the past 12 months? (2) How much time did you spend gambling on a typical day in which you gambled in the past 12 months? (3) How often did you spend more than 2 h gambling (on a single occasion) in the past 12 months?

#### 2.3.3. Alcohol

The Alcohol Use Disorders Identification Test consumption questions (AUDIT-C, [40]) are a subset of questions from a clinical screening tool to assess the misuse of alcohol: the AUDIT. The AUDIT-C covers only questions on alcohol consumption and is not significantly different to the full AUDIT [41] in identifying alcohol misuse. It also outperforms the CAGE screen [42,43], an alternative brief four question screen often used in primary care. The AUDIT-C consists of the following three questions: (1) In the past year, how often did you have a drink containing alcohol? (2) How many drinks containing alcohol did you have on a typical day when you were drinking? (3) Considering all types of alcoholic beverages, how often did you have six or more drinks on one occasion in the past year?

#### 2.3.4. Sleep

Self-perceived sleep quantity was assessed using the following question: During the past 30 days, for about how many days have you felt you did not get enough rest or sleep? This self-report question is commonly used in the literature to assess sleep behaviour [44,45,46]. The 30-day frame of reference for the question avoids the limitations of post-sleep questions which tend to be less reliable by assessing only the previous night’s sleep [46,47]. It has also been demonstrated to have good test/retest reliability [48]. Sleep problems were assessed using three questions from the DSM-V criteria for insomnia [49] that focused on the typical components of insomnia. These questions make up the insomnia component of the World Health Organisation Composite International Diagnostic Interview (WMH-CIDI, [50]). The three questions for insomnia are: Have you experienced the following symptoms for periods lasting 2 weeks or longer in the past 12 months (yes/no): (1) Nearly every night it took you two hours or longer before you could fall asleep? (2) You woke up nearly every night and took an hour or more to get back to sleep? (3) You woke up nearly every morning at least two hours earlier than you wanted to?

### 2.4. Statistical Analysis

All analyses were done using the IBM SPSS v26 software package. The relationships between total scores on the alcohol, gambling, and sleep variables across each of the three datasets (2015, 2016, 2016/2) were examined using Spearman’s Rho correlations.

Simultaneous multiple regression was used to assess the ability of CSPG, Lie/Bet, and AUDIT-C scores to predict insomnia and subjective sleep quality, controlling for the influence of the covariates of age and gender.

## 3. Results

### 3.1. Sample

As reported in Table 1, the 2015, 2016, and 2016/2 datasets consisted of 1318, 1217, and 1225 completed surveys, respectively. The response rate was between 26% and 35%, which aligns with comparable national telephone surveys at the time [51]. Demographic characteristics of each sample are presented in Table 2. The distribution across all major demographics in each of the three samples was comparable, including the percentage of males and females and split across age groups.

### 3.2. Relationships between Variables

Table 3 presents the correlations between the scores on the AUDIT-C, Lie/Bet, insomnia screen, CSPG, and a sleep quantity measure, across each of the three datasets. As expected, there was a significant positive correlation between scores on the Lie/Bet and the CSPG in all three datasets. There was also a significant positive correlation between insomnia and subjective sleep quality. Scores on the AUDIT-C, the Lie/Bet, and the CSPG were significantly positively correlated in all three datasets. In the dataset that included the insomnia screen, scores were significantly positively correlated with the Lie/Bet. The Lie/Bet was also significantly positively correlated with subjective sleep quality in two of the three datasets.

Multiple regression analysis results revealed that, in the 2015 dataset, the Lie/Bet score and AUDIT-C score were significant predictors of insomnia (see Table 4). In the 2016/1 dataset, the Lie/Bet score significantly predicted poorer subjective sleep quality. Moreover, gender and age were also shown to be significant predictors of subjective sleep quality in 2016/1 and 2016/2, whereby females nominated poorer sleep quality and older participants indicated relatively better sleep quality. For context, people in the prime working years (aged 35–44 years old) had 10–12 nights of poor sleep on average in the last 30 days, whereas people 55 and older had only 6–7 nights of poor sleep. Furthermore, male respondents averaged 7–8 nights of poor sleep, whereas female respondents indicated a significantly higher 9 nights in both 2016/1 and 2016/2. There was no significant relationship in sleep quality for people who were married or in a de facto relationship, and thus presumably co-sleeping with others.

## 4. Discussion

Few studies have examined the relationship between gambling, alcohol consumption, and sleep problems, despite the evidence that these latter factors negatively impact health and are likely to be exacerbated by the current gambling environment [8,9]. The current study addressed this gap in the literature by being the first large population study to examine the association between gambling, alcohol consumption, and sleep, using a representative national Australian sample. Both alcohol and gambling problems predicted insomnia, and gambling problems predicted poorer subjective sleep quality, although this latter relationship was only found in one dataset. We also found that more severe gambling problems were correlated with more severe alcohol use problems and insomnia symptoms, and were negatively associated with perceived sleep quality. The effect sizes found in the current study were relatively small, particularly for the sleep and gambling measures. However, these findings align with previous studies that examine the relationships between these factors in various combinations [21,23,34]. Nonetheless, contrary to previous literature, the current study did not find a relationship between alcohol misuse and subjective sleep quality, only alcohol misuse and insomnia. It is likely that this is due to the insomnia screen capturing individuals with more severe sleep problems and it is those that are related to alcohol misuse, rather than simply poor subjective sleep quality.

The present study used multiple measures of gambling and sleep problems, which allowed for slightly different aspects of these two factors to be investigated, using both a sleep disorder screen and a general sleep quality screen to detect clinical as well as less severe sleep problems. Both insomnia and self-perceived poor sleep quality were associated with gambling problems and when put into a predictive model, gambling and alcohol problems predicted insomnia and self-perceived sleep quality, adding weight to our findings. We also used two screens to measure gambling problems, and self-reports from both the CSPG and the Lie/Bet were positively associated with alcohol misuse. However, only the Lie/Bet was associated with sleep problems. The CSPG measures gambling consumption (i.e., frequency and duration of gambling sessions), whereas the Lie/Bet taps into two key behaviours directly related to problem gambling (lying about one’s gambling and an increased urge to gamble more money). Whilst gambling consumption is strongly associated with harm from gambling [40,52], an affirmative answer to the Lie/Bet indicates a more severe gambling problem. Thus, it appears that sleep problems are associated with gambling problems as a mental health condition, known in clinical circles as disordered gambling, rather than with gambling consumption more generally. It should be noted that, due to the study methodology, we cannot assert the directionality of the relationship between gambling problems and sleep problems.

Our study findings have implications for the treatment of gambling problems. Encouraging clinicians to not only screen for alcohol misuse but also for sleep problems may enhance the range of therapeutic options available to people experiencing harm from gambling. Benefits have been found in reducing the severity and length of major depressive disorder (MDD) when comorbid insomnia is also treated, as opposed to focusing treatment only on MDD [53,54]. Hence, similar attention paid to insomnia symptoms may lead to an improvement in gambling problems, although future studies may be needed to confirm this in a clinical setting. The higher incidence of poor sleep quality in females and those aged 34–44 years also indicates that it may be of clinical benefit to focus on screening this group for comorbid conditions.

The association between gambling problems, alcohol consumption, and sleep problems also has policy implications. Our findings highlight the potential risk of providing gambling in venues where alcohol is served, and opening hours are long. Venue staff are required to adhere to responsible gambling codes of conduct that include monitoring gambling patrons’ intoxication levels and their length of play [55,56,57]. In reality, interventions by venue staff are rare and, in some cases, actively discouraged [58]. The current research adds to the evidence that further restrictions to gambling venue opening hours and in-venue alcohol consumption may reduce harm from gambling [59,60,61]. The current study evidence also supports the development of innovative harm reduction methods for reducing harm from online gambling, which is a form of gambling that necessarily has few restrictions in terms of hours of access and the responsible service of alcohol [62]. However, studies investigating causation are needed before policy changes can be recommended.

A limitation of cross-sectional studies, including the current study, is that we are not able to infer direct causal links between each of these variables. Laboratory studies have established that alcohol causes changes in gambling behaviour [61,63], but few controlled laboratory studies have examined the link between sleep restriction and gambling [64]. Future research should utilise controlled laboratory experiments to identify whether sleep restriction may cause changes in gambling behaviour and whether gambling late into the night has a causal effect on sleep. In addition, so-called ‘third’ variables that may mediate the relationship between gambling, alcohol consumption, and sleep were not examined in this study. Examples of such third variables may be psychological problems, e.g., depression [10,65], emotional dysregulation [33], or traits such as impulsivity [66] or consumptiveness [67]. Future research should investigate these likely third variables in prospective or experimental design to isolate the contribution of each variable to the relationship between gambling, alcohol consumption, and sleep.

## 5. Conclusions

While there are some inconsistencies in the findings with respect to self-perceived sleep deficits, there is evidence that gambling problems and alcohol problems predict poor sleep, particularly symptoms of insomnia. Further research, such as controlled laboratory studies, is needed to understand whether there is a causal connection between gambling issues and sleep or whether these are related for other reasons. This study represents a unique contribution to the gambling literature in investigating the link between gambling, alcohol consumption, and sleep in a large representative sample, informing future research on the impact that other behaviours may have on gambling harm.

## Figures and Tables

**Table 1:** Sampling characteristics.

Year	Data Collection Period	Mean Interview Length (mins)	Number of Participants	Response Rate (%)
2015	6 July–14 Aug	33	1318	33
2016	13 June–2 Aug	42	1217	26
2017	2–29 Nov	25	1225	35

**Table 2:** Participant demographic information.

Characteristic	2015	2016	2016/2
Gender Male Female	602 (45.7) 716 (54.3)	603 (49.5) 614 (50.5)	584 (47.7) 641 (52.3)
Age, years 18–34 35–44 45–54 55+	255 (19.3) 186 (14.1) 219 (16.6) 645 (48.9)	239 (19.6) 174 (14.3) 194 (15.9) 600 (49.3)	217 (17.7) 156 (12.7) 204 (16.7) 640 (52.2)
Marital status Married/de facto Separated/divorced Widowed Single	877 (66.5) 103 (7.8) 81 (6.1) 245 (18.6)	795 (65.3) 122 (10.0) 68 (5.6) 225 (18.5)	801 (65.4) 115 (9.4) 70 (5.7) 232 (18.9)
Country of birth Australia Other	989 (75.0) 326 (25.0)	878 (72.1) 339 (27.9)	898 (73.3) 327 (26.7)
Highest level of education (complete or incomplete) Primary schooling or below Secondary/high school Technical studies or further education University or other higher education	26 (1.9) 410 (31.1) 292 (22.2) 583 (44.2)	22 (1.9) 342 (28.1) 294 (24.2) 556 (45.7)	23 (1.9) 360 (29.4) 277 (22.6) 560 (45.7)
Employment status Employed full-time Employed part-time/casual Unemployed Retired/pensioner Student Home duties	496 (37.6) 276 (21.0) 61 (4.6) 384 (29.2) 35 (2.7) 57 (4.3)	467 (38.4) 289 (23.7) 45 (3.7) 347 (28.5) 25 (2.1) 38 (3.1)	462 (37.7) 262 (21.4) 48 (3.9) 372 (30.4) 28 (2.3) 40 (3.3)
State or territory residing Australian Capital Territory (ACT) New South Wales (NSW) Northern Territory (NT) Queensland (QLD) South Australia (SA) Tasmania (TAS) Victoria (VIC) Western Australia (WA)	33 (2.5) 380 (28.8) 28 (2.1) 271 (20.6) 89 (6.8) 35 (2.7) 347 (26.3) 131 (9.9)	30 (2.5) 390 (32.0) 10 (0.8) 267 (21.9) 77 (6.3) 18 (1.5) 319 (26.2) 106 (8.7)	20 (1.6) 369 (30.1) 13 (1.1) 267 (21.8) 95 (7.8) 26 (2.1) 318 (26.0) 116 (9.5)

**Table 3:** Spearman’s Rho correlations between all variables across each dataset.

	Insomnia	Sleep Quality	AUDIT-C	CSPG	Lie/Bet	Dataset
Insomnia						
Sleep quality	0.33 * - -					2015
AUDIT-C	0.06 - -	0.00 0.00 0.05				2015 2016/1 2016/2
CSPG	0.08 - -	−0.09 −0.01 0.04	0.15 * 0.14 ** 0.18 **			2015 2016/1 2016/2
Lie/Bet	0.21 ** - -	0.01 0.06 * 0.10 **	0.17 ** 0.11 ** 0.08 **	0.22 ** 0.42 ** 0.34 **		2015 2016/1 2016/2

** Correlation is significant at the 0.01 level (2-tailed). * Correlation is significant at the 0.05 level (2-tailed).

**Table 4:** Regression analysis for predictors of insomnia score (2015), and subjective sleep quality (2015, 2016; 1 & 2).

	Insomnia (2015)			Sleep Quality (2015)			Sleep Quality (2016/1)			Sleep Quality (2016/2)		
Variable	B	SEB	β	B	SEB	β	B	SEB	β	B	SEB	β
Gender (female)	0.16	0.13	0.09	2.29	1.32	0.12	1.76	0.64	0.09 **	1.73	0.61	0.08 **
Age	0	0	0.07	−0.05	0.04	−0.9	−0.09	0.02	−0.16 **	−0.13	0.02	−0.22 **
Married/ de-facto	−0.03	0.07	−0.03	−0.02	0.69	0	−0.37	0.33	−0.04	−0.07	0.31	−0.01
AUDIT-C score	0.06	0.03	0.16 *	0.30	0.26	0.08	0.04	0.15	0.01	−0.09	0.14	−0.02
CSPG score	0	0.03	0.01	−0.26	0.28	−0.7	−0.24	0.26	−0.03	0.12	0.2	0.02
Lie/Bet score	0.36	0.13	0.19 **	0.97	1.26	0.05	3.74	1.51	0.09 *	2.29	1.37	0.05

** Correlation is significant at the 0.01 level (2-tailed). * Correlation is significant at the 0.05 level (2-tailed).

## Data Availability

The data is not publicly available, although we would allow access on request to people who wish to verify our results.
